# Beyond Polymer Identity: Biological and Health Implications of Polyvinyl Chloride Micro- and Nanoplastics

**DOI:** 10.3390/molecules31213826

**Published:** 2026-10-23

**Authors:** Adrian Kawecki, Magdalena Pietrzak, Marek Jutel, Magdalena Zemelka-Wiacek

**Affiliations:** Department of Clinical Immunology, Faculty of Medicine, Wroclaw Medical University, 51-616 Wroclaw, Poland

**Keywords:** polyvinyl chloride, microplastics, nanoplastics, human exposure, human biological samples, biological interfaces, cellular and tissue responses, particle toxicology, One Health

## Abstract

Polyvinyl chloride (PVC) micro- and nanoplastics (PVC-MNPs) represent heterogeneous exposures shaped by product formulation, ageing, environmental transformation and the release of associated chemicals. This review examines evidence on human exposure, tissue distribution and biological effects of PVC-MNPs, emphasising biological barriers and potential health implications. Exposure may occur through ingestion, inhalation, dermal contact and medical devices, while PVC has been detected in blood, placenta, reproductive and respiratory samples, and other human tissues. Experimental studies indicate that PVC-MNPs can impair epithelial homeostasis and induce oxidative stress, inflammatory responses, mitochondrial dysfunction and metabolic alterations. However, these effects vary with particle size, formulation, concentration, ageing, biological conditioning and experimental model. Environmental and biological transformations may modify particle properties, chemical release and microbial interactions, linking environmental contamination with food systems and human exposure within a One Health framework. Interpretation of the available evidence is limited by methodological heterogeneity, contamination risks, uncertain exposure doses, and limited comparability between experimental and clinical studies. Current evidence supports internal exposure and the biological plausibility of cellular and tissue injury but does not establish a causal relationship between PVC-MNP exposure and human disease. Future studies should integrate source-resolved exposure assessment, physiologically relevant models, appropriate leachate controls and standardised analytical methods.

## 1. Introduction

Microplastics (MPs) and nanoplastics (NPs) are particles generated throughout the life cycle of polymer-containing products, including their manufacture, use, ageing and fragmentation. MPs are generally defined as plastic particles from 1 µm to 5 mm, whereas NPs represent the smaller fraction below 1 µm [1]; here, both are referred to collectively as micro- and nanoplastics (MNPs). The exposome, which encompasses all non-genetic exposures throughout life, provides a framework for understanding the relevance of MNPs to human health. The One Health framework links environmental, animal and human health. MNPs coexist with indoor and outdoor air pollutants, allergens, microbial products and other chemical or biological exposures that may shape epithelial and immune responses [2,3,4,5].

Research on MNPs and human health now extends beyond environmental occurrence to their detection in human tissues. A growing number of studies have examined the potential biological effects of MNPs. However, much of the current knowledge comes from in vitro studies, which cannot determine whether these particles cause human disease. Such models provide valuable mechanistic insights, yet they do not reproduce the full complexity of organismal exposure, including absorption, distribution, repeated low-dose exposure, metabolism, clearance and interactions between tissues [6,7,8,9,10,11]. At the same time, animal studies are extending the evidence beyond cell-based systems by examining integrated intestinal, reproductive, systemic and respiratory responses following exposure to polyvinyl chloride (PVC) micro- and nanoplastics (PVC-MNPs) [12,13,14,15]. Human biomonitoring studies have additionally reported MNPs, including PVC in selected analyses, in coronary blood, upper-airway samples, placental tissue, testes and kidney-associated donor tissues [16,17,18,19,20]. Human data suggest an association between MNPs in atherosclerotic plaques and cardiovascular events. A prospective observational study of patients undergoing carotid endarterectomy detected polyethylene (PE) in 58.4% of analysed plaques and PVC in 12.1%. The presence of MNPs in plaques was associated with a higher hazard of the composite outcome of myocardial infarction, stroke or death from any cause during follow-up (adjusted hazard ratio, 4.53; 95% confidence interval, 2.00–10.27). This association concerned MNP detection overall and does not provide a PVC-specific estimate of cardiovascular risk [21].

PVC-MNPs should not be interpreted as particles consisting only of a chemically uniform PVC polymer. PVC is a thermoplastic polymer composed of repeating vinyl chloride units, with chlorine atoms attached to the carbon backbone. This distinguishes it from hydrocarbon polymers such as PE or polypropylene (PP). Commercial PVC usually contains additives in addition to the polymer. Depending on application, PVC may be produced as rigid or plasticised material and may contain plasticisers, heat stabilisers, pigments, fillers, flame-retardant components and metal-based additives. Particles released from pipes, flooring, cable insulation, medical tubing, packaging or flexible coated products may therefore differ in morphology, additive burden and chemical release [22,23,24]. For this reason, PVC-MNPs may expose living organisms not only to polymer particles, but also to leachates. Leachates are liquids containing chemicals released from plastic particles or products into surrounding media, such as water, gastrointestinal fluids or cell-culture medium. In PVC, leachates may include plasticisers, degradation products and other formulation-derived compounds. Photoageing of PVC-MNPs increases the release of di(2-ethylhexyl) phthalate (DEHP) and its transformation products, indicating that an aged PVC fragment may represent a different exposure than a pristine laboratory particle [25]. Digestive conditions and contact with biomolecules may further modify particle behaviour and chemical bioaccessibility [26,27].

From a human-health perspective, the biological effects of PVC-MNPs depend first on the route of exposure. Following oral exposure, PVC-MNPs first encounter the luminal contents of the gastrointestinal tract and subsequently interact with the mucus layer, intestinal epithelium, microbiota and underlying gut-associated immune system. Following inhalation, particles are deposited within the respiratory tract, where they contact the airway mucus, respiratory epithelium and resident immune cells. Following dermal exposure, particles initially contact the skin surface and stratum corneum; penetration into viable skin and systemic absorption require separate evidence. During intravenous infusion, PVC-MNPs released from medical devices may contact blood and circulating plasma proteins directly, bypassing epithelial entry barriers. Environmentally sourced PVC increased colon inflammation and fibrosis in mice, particularly under chronic colitis conditions [28]. In a human induced pluripotent stem cell-derived intestinal epithelium model, true-to-life MPs, including PVC, impaired barrier function and induced oxidative and cytokine responses; their toxicity correlated with protein corona composition [10]. After contact with biological fluids, MNPs adsorb proteins and other biomolecules onto their surface, forming a protein corona that changes the particle’s biological identity. This corona may modify aggregation, surface chemistry, cellular uptake, immune recognition and toxicity. Consequently, a particle tested in water is not biologically equivalent to the same particle after contact with intestinal fluid, plasma or airway-lining fluid [29,30,31,32,33,34].

This review interprets PVC-MNPs through a framework that distinguishes particle-associated effects, effects of released chemicals, and responses to their combined exposure. These components are considered alongside product formulation, ageing, biological conditioning and exposure route. The framework is used to compare experimental findings, evaluate their relevance to human exposure, and connect environmental and animal evidence with potential human-health implications. Throughout, responses to PVC-containing test materials are distinguished from mechanisms attributable specifically to the PVC polymer.

## 2. Literature Search Strategy

A structured, iterative literature search was conducted to support this focused narrative review of PVC-MNPs in relation to human exposure, tissue distribution, biological effects, material transformation and methodological interpretation. PubMed, Scopus, Web of Science (WoS) and Embase were searched in May 2026. The core search combined “polyvinyl chloride” or “PVC” with “microplastic*” or “nanoplastic*”. Database-specific searches additionally incorporated terms related to toxicity, inflammation, oxidative stress, gastrointestinal, respiratory and dermal exposure, medical-device exposure, human biomonitoring and biological samples. The four database exports contained 1677 records, which were deduplicated in Zotero (version 10.0.6; https://www.zotero.org/, accessed on 1 October 2026) to 845 unique records. English-language studies published primarily from 2020 onwards were prioritised, while earlier publications were retained when they provided relevant physiological, material or methodological evidence.

Sources were selected iteratively according to their relevance to PVC sources and formulation, particle ageing and chemical release, human exposure routes, detection in human biological samples, experimental biological effects, environmental processes and analytical limitations. Original human, animal, cellular and environmental studies were prioritised for reporting specific findings, while reviews and methodological publications were used to support definitions, physiological context and interpretation. Because this was a focused narrative review, source selection was relevance-based and was not conducted according to a formal systematic-review or meta-analysis protocol.

Polymer attribution is assessed at the level of each reported finding rather than assigned to an entire study. We distinguish: (i) PVC identification or quantification in environmental and biological samples; (ii) experimental responses or observational associations evaluated separately for PVC; and (iii) aggregate findings concerning multiple polymers, including studies in which PVC is detected but the reported outcome relates to total MNP burden. Evidence concerning other polymers is retained for physiological, methodological or comparative context, with its scope explicitly stated. Identifying PVC in a sample does not, by itself, attribute a biological outcome to that polymer. Likewise, a response to a PVC test material does not establish a mechanism unique to the PVC polymer.

Evidence was interpreted using the qualitative framework described in Section Evidence Strength and Inferential Limits. We distinguished occurrence in biological specimens, human observational associations, experimental effects and mechanisms, and human disease causation. Evidential strength was considered within each category, taking into account analytical reliability, material and dose characterisation, study design, biological relevance and consistency across studies. This narrative appraisal did not assign numerical quality scores or apply a formal systematic-review risk-of-bias instrument.

**Figure 1:**
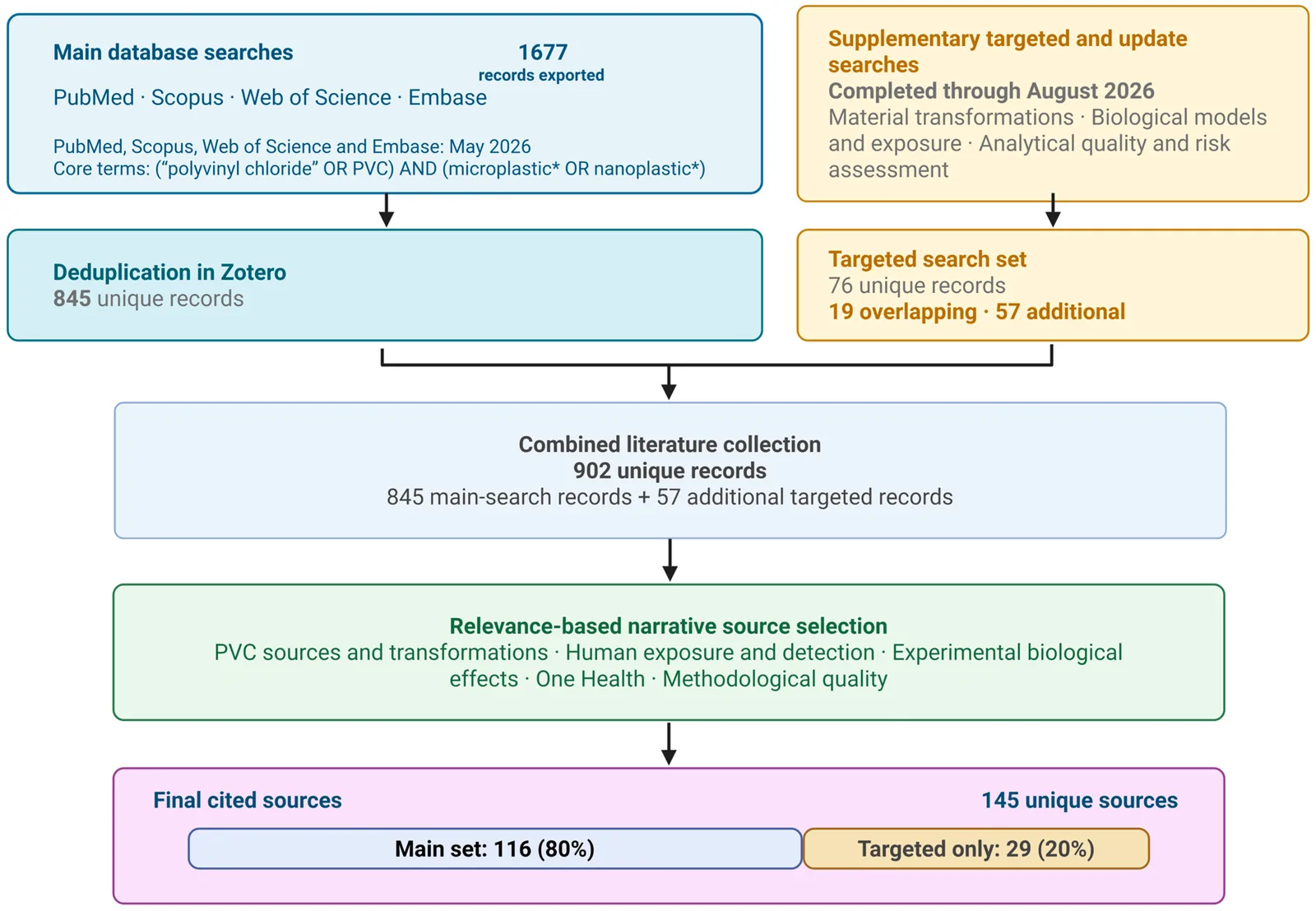
Literature search and relevance-based source-selection workflow for this focused narrative review. The main database searches yielded 1677 records, reduced to 845 unique records after deduplication. Supplementary targeted and update searches yielded 76 unique records, including 19 already present in the main search set and 57 additional records, resulting in a combined collection of 902 unique records. The manuscript cites 145 unique sources: 116 were present in the main deduplicated search set and 29 were identified exclusively through targeted searches. Sources retrieved through both routes were counted once. In the search terms, the asterisk (*) denotes a truncation wildcard. Abbreviations: PVC, polyvinyl chloride. Created in BioRender. Kawecki, A. (2026) https://BioRender.com/m46ox27, (accessed on 20 October 2026).

Supplementary targeted and update searches were subsequently conducted through August 2026 to address specific evidence gaps. These searches covered PVC photoageing and leachate release, protein corona formation, respiratory and dermal models, medical-device and intravenous exposure, One Health and ecotoxicological evidence, exposure- and risk-assessment frameworks, analytical quality assurance and contamination controls, and reference and test materials for micro- and nanoplastic research. The targeted-search set comprised 76 unique records, including 19 already present in the main search set and 57 additional records, yielding a combined collection of 902 unique records. The current manuscript cites 145 unique sources: 116 were present in the main deduplicated search set, and 29 were identified exclusively through targeted searches. Sources retrieved through both routes were counted once. The literature-search and source-selection workflow is summarised in Figure 1.

## 3. PVC-Based Products as Sources of MNPs

**Figure 2:**
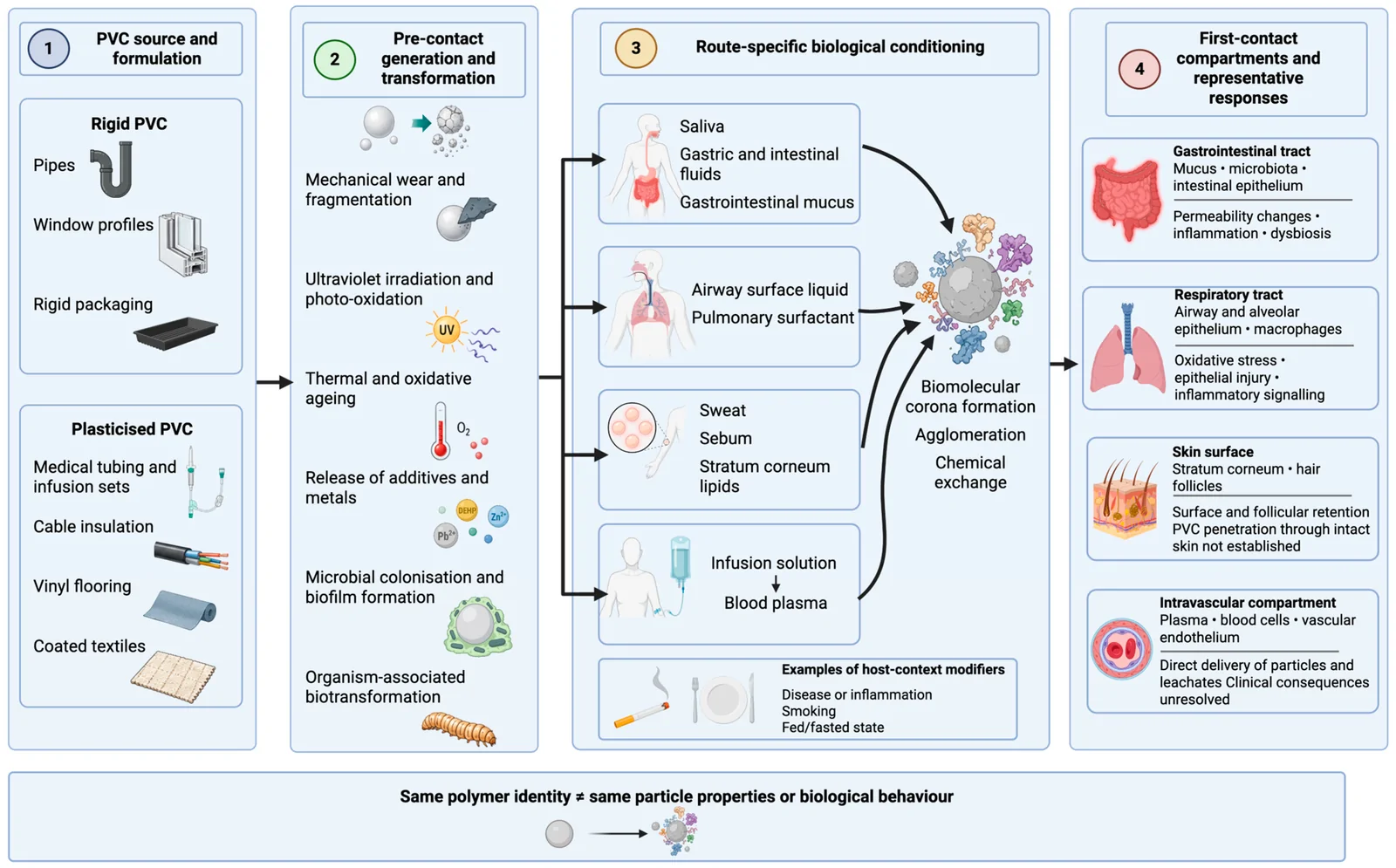
Source-to-first-contact pathway of polyvinyl chloride micro- and nanoplastics (PVC-MNPs). Product formulation and pre-contact transformations influence particle properties and the release of associated chemicals. Route-specific biological media may further modify particle agglomeration, biomolecular corona formation and chemical exchange before contact with gastrointestinal, respiratory, cutaneous or intravascular compartments. The responses shown are representative and do not imply equivalent evidence across exposure routes or causation in humans. Abbreviations: DEHP, di(2-ethylhexyl) phthalate; PVC, polyvinyl chloride; PVC-MNPs, polyvinyl chloride micro- and nanoplastics; UV, ultraviolet. Created in BioRender. Kawecki, A. (2026) https://BioRender.com/hi1ysm2, (accessed on 20 October 2026).

For PVC-MNPs, polymer identification alone does not fully characterise exposure; product origin and the transformations occurring before biological contact also determine particle properties. In drinking-water systems, micromolar residual disinfectants aged unplasticised PVC pipe particles and increased the release of organic matter, including plasticisers and antioxidants [35]. After experimental high-temperature steam disinfection, disposable medical-device samples released mean particle numbers ranging from (1.08 ± 0.14) × 10^5^ for a PVC device to (1.27 ± 0.34) × 10^6^ for a PP device. The mean sizes of the released particles were 11.45 ± 1.79 µm and 7.18 ± 0.52 µm, respectively [36]. Particle origin and morphology also influenced biological effects. In marine medaka, commercially obtained PVC powder particles, classified as primary MPs, were compared with irregular secondary MPs produced by milling a pristine PVC water pipe; both fractions were sieved to 53–106 µm [37]. The secondary particles caused greater embryotoxicity, mainly through physical damage to the chorion, the protective membrane surrounding the embryo [37]. Photoageing in the presence of humic acid (natural organic matter from decomposition) increased the acute toxicity and genotoxicity of leachates from PVC MPs under light irradiation [38]. More broadly, ageing-related changes in polymer structure and surface area can enhance additive migration and leaching [39] (Figure 2).

PVC-based medical devices represent a distinct exposure scenario because PVC-MNPs released into infusion solutions could be delivered directly into the circulation, bypassing epithelial barriers [40,41]. In eight marketed devices intended for infusion and enteral or parenteral nutrition, plasticisers accounted for 25–36% of PVC mass, illustrating the substantial plasticiser content of these devices [42]. New disposable PVC infusion tubes already carried an average of 931.4 PVC microplastics (PVC-MPs) and 0.040 µg of PVC nanoplastics (PVC-NPs) per tube. After four hours at room temperature with 0.9% sodium chloride, 5% glucose, 5% sodium bicarbonate or hydroxyethyl starch 40 in sodium chloride, the corresponding quantities ranged from 1003.6 to 3494.6 PVC-MPs and from 0.042 to 0.087 μg of PVC-NPs, and increased further with exposure duration and temperature [40]. In a separate study, passage of 0.9% sodium chloride through infusion tubes for four hours at room temperature yielded approximately 16 MPs/mL, mainly polyamide, PVC and polyethylene terephthalate (PET), with higher release at elevated temperatures [41]. Both studies reproduced device use under laboratory conditions; the particle dose actually administered to patients, subsequent retention and clinical consequences were not determined.

Additives are a defining feature of PVC-based particles. Plasticisers reduce PVC stiffness by increasing mobility between polymer chains, which allows the production of flexible materials. Plasticisers used in PVC differ in molecular structure and physicochemical properties [23,42], while their migration or release reflects interactions with the polymer matrix and varies with particle size, temperature and the composition of the surrounding medium [43,44]. Phthalate plasticisers contribute to the chemical exposure associated with PVC-MNPs because they are not covalently bound to PVC and may therefore migrate into surrounding media. In a modified in vitro gastrointestinal extraction protocol, phthalates migrated from PVC during salivary, gastric, duodenal and biliary phases; inclusion of digestive enzymes reduced the migrated mass from 0.301 ± 0.05 to 0.226 ± 0.04 μg, consistent with the formation of an enzyme coating on the plastic surface [45]. In a separate physiologically based extraction study, bioaccessible fractions of moderately to non-polar phthalates released from PVC and low-density PE MPs ranged from 1.8% to 32.2% and were highest under fed-state gastrointestinal conditions, although they were generally greater for low-density PE than for PVC [46]. For DEHP, decreasing particle size enhanced its release, while increasing the temperature from 15 °C to 45 °C increased release from PVC MPs by 109%; increasing the sodium-ion concentration from 0 to 200 mM reduced it by 35.3% [43]. Thus, chemical exposure associated with PVC-MNPs depends on particle dimensions and surrounding conditions, rather than solely on polymer identity.

Metal-containing stabilisers, pigments and other inorganic additives further increase the heterogeneity of PVC-MNPs. After accelerated chemical ageing of commercial PVC MPs with 30% hydrogen peroxide (H_2_O_2_), the fractions of the total metal content released were 87.4% for chromium (Cr), 79.3% for nickel (Ni), 22.0% for lead (Pb), 20.9% for copper (Cu), 17.1% for zinc (Zn), 15.1% for cadmium (Cd) and 11.0% for manganese (Mn). Despite its lower proportional release, the absolute mass of released Pb exceeded that of the other metals by one to two orders of magnitude [47].

In beached marine plastics, including PVC samples, Pb present as an additive or manufacturing residue showed lower proportional bioaccessibility in an avian physiologically based extraction test than surface-adsorbed Pb (up to 16% versus 60–70%). However, because total Pb concentrations in additive-containing plastics reached 40,000 µg/g, their bioaccessible Pb concentrations exceeded those associated with adsorbed Pb by several orders of magnitude [48]. Thus, the metal burden of PVC particles may reflect the original formulation and residues introduced through recycled material, as well as subsequent environmental sorption [47,48].

Plastic ageing comprises physical, chemical and biotic processes that modify particle dimensions, morphology, polymer structure, surface chemistry and the mobility of associated chemicals before human contact. Depending on the ageing pathway, these changes may include oxidation, chain scission, dehydrochlorination, cracking, fragmentation, additive loss and altered sorption or leaching behaviour [49,50].

In PVC MPs, experimental ultraviolet (UV) irradiation increased surface roughness and cracking, the abundance of oxygen-containing functional groups, the carbonyl index (a spectroscopic measure of oxidation-related carbonyl groups), crystallinity and reactive oxygen species (ROS) formation with ageing time [51]. After 30 days of UV exposure, the adsorption capacity of PVC MPs for hydrophobic chlorpyrifos decreased, whereas adsorption of moderately hydrophilic erythromycin increased [52]. After 48 days of dry UV photoageing, the continuous DEHP leaching rate increased 1.5-fold, the estimated leaching half-life decreased from 449 to 121 years, and mono(2-ethylhexyl) phthalate (MEHP), phthalic acid and phthalic anhydride were identified as transformation products [25].

Biotic transformation includes surface colonisation and, in selected experimental systems, organism-associated microbial attack on the polymer matrix. In freshwater flow-through experiments, adenosine triphosphate (ATP) was used to quantify biofilm abundance; ATP levels on PVC were 6–12-fold higher than on the other tested polymers [53]. In *Tenebrio molitor* larvae fed rigid PVC MPs, the weight-, number- and size-average molecular weights of the residual polymer decreased by 33.4%, 32.8% and 36.4%, respectively, while chloride release represented approximately 2.9% of ingested PVC. Suppression of the gut microbiota with gentamicin markedly inhibited depolymerisation [54]. These findings demonstrate limited, microbiota-associated PVC transformation under specific laboratory conditions rather than rapid degradation of environmental PVC.

These source and transformation data motivate the interpretive framework used throughout this review. Particle-associated effects concern interactions involving the particulate material, including surface contact, uptake and responses influenced by particle dimensions, morphology and biological conditioning. Chemical effects concern substances released from that material, including additives and transformation products; photoageing-dependent DEHP release provides a documented PVC example [25]. Combined exposure involves both components, but their coexistence does not establish an additive or synergistic interaction. In zebrafish, comparisons of separate and combined PVC-MP and DEHP exposures identified antagonistic responses for cardiac-development gene expression [55]. This co-exposure experiment informs mixture interpretation but does not directly reproduce chemical release from a formulated particle.

Selected experiments illustrate why these components should be distinguished. In marine medaka embryos, differences between primary and secondary PVC particles were attributed predominantly to physical interactions with the chorion, with negligible chemical contribution under the tested conditions [37]. In a freshwater detritivore system, Ockenden et al. compared PVC MPs without added chemicals, dibutyl phthalate (DBP) alone and PVC with incorporated DBP. PVC without added chemicals produced limited effects, including reduced feeding at the higher tested concentration, whereas DBP-containing exposures affected respiration, feeding and assimilation [56]. This contrast supports an important contribution of released additives in that system. Neither finding establishes a universally dominant mechanism across PVC materials or biological systems. Where matched particle and leachate comparisons are unavailable, the appropriate inference concerns the tested PVC-containing material as a whole; attribution to particle properties, released chemicals or their interaction remains unresolved.

## 4. Human Exposure Routes: Ingestion, Inhalation, Skin and Medical Contact

Human exposure to PVC-MNPs may occur through ingestion, inhalation, dermal contact and medical-device use. These routes differ in the initial anatomical site and biological medium encountered [57,58]. The exposure pathways and corresponding first-contact compartments are summarised in Figure 2.

Ingestion may occur through drinking water, food and inadvertent swallowing of settled indoor dust. MPs were detected in all 177 tap-water samples collected across 13 cities in the United Kingdom, at concentrations of 6–100 particles/L, and in all 85 bottled-water samples, at 12–62 particles/L. PVC was among the three most frequently detected polymers in tap water, whereas PE, PP and PET predominated in bottled water [59]. In a mass-based analysis using pyrolysis–gas chromatography–mass spectrometry, PVC was detected in every analysed edible-tissue sample from oysters, prawns, squid, crabs and sardines; this method quantified polymer mass rather than individual particle number [60]. In three commercially important fish species from the Bay of Bengal, dried products contained significantly more MPs than corresponding fresh fish, while PVC represented 16% of the identified polymers [61]. Settled indoor dust provides an additional oral pathway, particularly through hand-to-mouth transfer. In dust from 30 homes and 30 workplaces in Birmingham, PVC was the third most abundant polymer, while estimated typical daily ingestion of total MPs was 103 particles/day in toddlers and 56 particles/day in adults [62].

The occurrence of PVC in indoor and outdoor air supports inhalation as a plausible exposure route. Active sampling in 30 homes and 30 workplaces in Birmingham measured mean total MP concentrations of 15.6 ± 5.4 and 13.1 ± 6.5 particles/m^3^, respectively. PET and PVC were the most abundant polymers, and particles measuring 10–25 µm predominated in the actively collected fraction [63]. In atmospheric particulate matter with an aerodynamic diameter of 2.5 µm or less from Shenyang, MNPs were detected in every sample at a mean mass concentration of 7.62 µg/m^3^, with PE and PVC as the predominant polymers [64]. In Marikina, total MP concentrations were higher outdoors than indoors (0.341 versus 0.155 particles/m^3^), whereas PVC accounted for 50% of the polymer identifications in indoor samples [65]. Airborne concentration is not equivalent to the dose deposited or retained in the respiratory tract. Deposition depends on aerodynamic diameter, particle shape, breathing pattern and airway geometry; coarser particles are preferentially retained in the upper and conducting airways, whereas smaller respirable particles have greater potential to reach distal bronchiolar and alveolar regions [66,67]. Material deposited in the conducting airways may subsequently be transported towards the pharynx by mucociliary clearance and swallowed. This creates secondary gastrointestinal exposure, while inhalation remains the initial route of entry [68,69].

Dermal contact refers to particle deposition or retention on the skin surface and does not itself demonstrate penetration into the viable epidermis or systemic absorption. In a study of ten personal-care and cosmetic products, PE was identified in nine products, while spectral bands consistent with PVC and PET were reported in some samples. The tested products included toothpaste, face wash, facial and body scrubs, a mask, nail polish and eyeliner [70]. Commercial glitter is a composite microplastic powder containing polymeric material and pigments. A chemical-release study compared finished glitter with PE and PVC used in its production. Incubation in artificial sweat released only low concentrations of metals and metalloids, but the experiment assessed chemical desorption rather than penetration of the plastic particles themselves [71]. Evidence concerning particle penetration through skin is derived mainly from non-PVC model polymers. A review of dermal exposure literature reported that most experiments used fluorescently labelled polystyrene (PS) particles and generally reported particle retention in the stratum corneum or hair follicles, with limited evidence of penetration into deeper tissues [72]. In an ex vivo human-skin model, most 100 nm PS particles remained on the surface when the stratum corneum was intact, whereas removal of this layer allowed particles to enter all layers of the epidermis [73]. Because the available skin-penetration studies are predominantly based on PS, direct penetration of PVC-MNPs through intact human skin remains unproven [72,73].

Medical-device-associated exposure differs from environmental exposure because intravenous administration and extracorporeal circulation bypass epithelial entry barriers and may introduce particles directly into the vascular compartment. In a simulated infusion study involving six types of commonly used intravenous infusion products, PP and PVC particles ranging from 10 nm to 3.6 µm were identified, with a mean number concentration of (5.82 ± 0.86) × 10^4^ particles/L [74]. In a second controlled study, glucose-containing infusion solutions generated a total MNP mass of 3.82 µg, which was 6.47 times that generated by distilled water (0.59 µg). Increasing the infusion volume from 500 to 2000 mL increased the released particle mass 2.02-fold; both effects predominantly involved PVC-MNPs [75].

In a prospective study of 22 children undergoing cardiac surgery, total blood MNP concentrations and PVC concentrations were significantly higher after cardiopulmonary bypass than before the procedure. Bypass duration was positively correlated with the increase in total blood MNP concentration (*r* = 0.43, *p* = 0.047) [76]. A separate study analysed 30 unopened haemodialysis concentrates and peritoneal-dialysis solutions and reported 36 suspected particles, predominantly fibres. PE, PVC and ethylene-vinyl acetate were among the principal synthetic polymers identified. Because the study did not assess completed dialysis circuits or patient blood, it does not demonstrate transfer of these particles into the systemic circulation [77]. Collectively, these findings identify intravenous infusion and extracorporeal procedures as clinically distinct potential routes of direct vascular exposure. However, patient-specific internal doses and clinical consequences remain unresolved.

## 5. Human Tissue Distribution and Clinical Associations

The analytical method determines what information can be obtained from human specimens. Micro-Fourier transform infrared spectroscopy (µFTIR), Raman microspectroscopy and laser direct infrared spectroscopy (LDIR) identify individual particles and can provide information on their polymer identity, size and morphology above technique-specific detection limits. By contrast, pyrolysis–gas chromatography–mass spectrometry (Py-GC/MS) reports polymer-specific mass from thermal degradation products without retaining information on particle number, morphology or tissue localisation [78]. In blood, the reliability of Py-GC/MS additionally depends on extraction recovery, matrix interference, marker selection and polymer-specific detection limits; PE and PVC require particular caution because interference and nonspecific pyrolysis products can complicate their quantification [79,80].

Multimodal workflows can provide complementary information on particle characteristics and polymer mass. For example, a study of human thrombi combined Py-GC/MS with LDIR and scanning electron microscopy to assess polymer mass concentrations alongside particle characteristics [81]. In a further cross-platform study of human endometrial and urine samples, Py-GC/MS detected additional polymer types, including PVC, that had not been identified by Raman microscopy under the study protocol [82]. This illustrates how complementary methods can reveal differences in the polymer profiles obtained from biological samples. Where sufficient specimen material is available, future biomonitoring studies should analyse matched aliquots from the same specimen, with documented homogenisation, subsampling and analytical coverage. This approach may help distinguish methodological differences from between-specimen variability, although heterogeneous particle distribution and differences in recovery remain important limitations. The resulting endpoints remain distinct: Py-GC/MS is destructive, and estimating polymer mass from particle counts requires explicit assumptions about particle dimensions, geometry and density. Results from different analytical methods should therefore be interpreted as complementary. Particle counts and polymer mass are not interchangeable.

Building on existing microplastic reporting guidelines and analytical validation studies, we propose a minimum reporting framework for human PVC biomonitoring [79,80,83]. This should include: (i) specimen type, anatomical sampling site, relevant participant characteristics, and analysed sample mass or volume; (ii) collection and storage materials, digestion and extraction procedures, filtration cut-offs, and contamination precautions; (iii) the effective size range of particle-based analysis and polymer-specific method limits of detection and quantification, including how these were determined; (iv) spectral libraries and identification thresholds, or pyrolysis markers and calibration procedures, with assessment of potential PVC-related matrix interference; (v) particle counts by polymer, size class and morphology, and polymer mass where measured, reported separately with explicit units and sample denominators, including whether tissue concentrations refer to wet or dry mass; and (vi) sampling and procedural blanks, blank-correction rules, matrix-specific recoveries, replicate variability, measurement uncertainty, and treatment of results below detection or quantification limits. The main quality assurance and quality control (QA/QC) concerns are contamination, matrix-dependent recovery, lower particle-size detection limits and polymer-specific limits of detection and quantification. Particle counts and polymer mass are distinct endpoints and should be reported separately. Blanks and recovery experiments are essential, and non-detection does not establish the absence of PVC from a specimen [79,80,83].

Blood and vascular specimens provide some of the most clinically detailed human data. Among 101 individuals presenting with chest pain, patients with acute coronary syndrome had higher total blood MP concentrations than controls, with the highest burdens observed in acute myocardial infarction. Total MP concentrations were positively associated with interleukin-6 (IL-6) and interleukin-12p70 (IL-12p70) concentrations and with B-cell and natural killer (NK)-cell counts. PVC was the second most abundant polymer after PE, but these immunoinflammatory associations were calculated for total MP burden rather than PVC separately [84]. MPs were detected in 24 of 30 thrombi retrieved from cerebral and coronary arteries and deep veins. Polyamide 66 (PA66; nylon 66), PVC and PE were identified; higher total MP concentrations were associated with greater disease severity, whereas D-dimer concentrations were higher in the MP-detected group than in the MP-undetected group [81]. In paired samples from 20 patients undergoing carotid endarterectomy, mean total MP concentrations were 75.2 µg/g in blood and 432.9 µg/g in plaque, and PVC represented 16.8% and 18.5% of the respective polymer burdens. The modest paired correlation for total MPs (*r* = 0.30) suggests only limited correspondence between circulating and plaque measurements [85].

Compartment-specific patterns are also apparent outside vascular lesions. In 229 blood and 227 faecal samples, PVC was among the most prevalent polymers in both specimens, but blood and faecal PVC concentrations were negatively correlated; drinking-water source was the strongest predictor of blood PVC according to the machine-learning models [86]. MPs were detected in all 16 bone-marrow samples analysed, with a mean total concentration of 51.29 µg/g, and PVC was present in 75% of samples [87].

Cerebrospinal fluid (CSF) studies provide evidence from a fluid surrounding the brain and spinal cord. In one study, CSF PVC and PE concentrations were higher in 17 amyloid-positive participants than in 15 amyloid-negative participants. Amyloid positivity refers to biomarker evidence of amyloid-β pathology, a feature associated with Alzheimer’s disease. However, only PE was associated with lower CSF amyloid-β42, lower scores on the Mini-Mental State Examination (MMSE), a test of global cognitive function, and further cognitive decline during follow-up [88]. In paired CSF and blood samples from 20 participants, PVC, PA66, PE, PP and PS were detected in both fluids, while total MNP concentrations were lower in CSF than in blood. Correlations between CSF and blood burdens occurred in the neuroimmune and neuroinfectious groups but not in controls, and the high-total-MNPs group showed changes in 23 metabolic pathways; these findings were not PVC-specific [89].

Bronchoalveolar lavage fluid (BALF) contains material recovered from the bronchiolar and alveolar compartments. In 30 patients undergoing bronchoscopy for diffuse lung disease, fluorescence-based screening detected a median of 684.7 suspected particles/mL, more than 80% of which were smaller than 10 µm. Raman spectroscopy of a subset of particles identified PVC, PS and PET as the most frequent polymers. Total particle concentration was positively correlated with serum C-reactive protein and was higher in lung regions showing radiological consolidation, in which alveolar air is replaced by fluid, exudate, inflammatory cells or other material. These associations were not analysed separately for PVC, and the clinically selected cohort limits their generalisability [90].

In a study of 62 placentas, Py-GC/MS detected plastic polymers in all samples, with PVC accounting for approximately 10% of the total polymer mass and PE predominating [91]. These mass-based measurements do not provide particle counts and do not, on their own, demonstrate foetal transfer or adverse pregnancy outcomes.

Three analyses of large pregnancy cohorts from Shenyang, comprising 1057–1324 participants, examined whether concentrations of PVC particles in placental tissue were associated with biomarkers measured in the placenta or umbilical cord blood at delivery. Higher placental PVC concentrations were associated with shorter telomeres (protective structures at chromosome ends that can shorten with cellular ageing or stress) in both placental tissue and cord blood [92]. Higher placental PVC concentrations were also associated with lower cord-blood cortisol and cortisone, which are closely related glucocorticoids involved in metabolism and the stress response, and with higher androstenedione, a precursor of androgen hormones [93]. A third analysis linked higher placental PVC concentrations specifically to increased cord-blood alkaline phosphatase (ALP) activity, one of four enzyme activities measured in the study. Associations with alanine aminotransferase (ALT), aspartate aminotransferase (AST) and gamma-glutamyl transferase (GGT) were observed mainly for PP or total MP burden rather than PVC [94]. These observational associations do not demonstrate that PVC particles crossed the placenta or caused the biomarker changes.

Human reproductive samples provide complementary clinical evidence. In follicular fluid and seminal plasma from 51 couples undergoing in vitro fertilisation, mean PVC concentrations were 1.85 and 2.67 µg/g, respectively. Higher follicular-fluid PVC was associated with lower fertilisation rates, and higher seminal-plasma PVC with reduced sperm motility; neither embryo implantation nor clinical pregnancy was associated with parental PVC levels [95]. PVC and PE were also the predominant polymers in semen from a separate study of 30 semen and six testicular samples, although PS predominated in testicular tissue [96]. An exploratory case-control study analysed follicular fluid from 25 women with diminished ovarian reserve (DOR) and 20 women with tubal-factor infertility serving as controls. DOR refers to a reduced pool of recruitable ovarian follicles and a lower expected response to ovarian stimulation. Total MP mass was higher in the DOR group (30.63 vs. 18.48 µg/g), and PE, PP and PVC concentrations were individually elevated. In multivariable analysis, higher follicular-fluid PVC remained associated with DOR and PVC concentration was negatively correlated with anti-Müllerian hormone (AMH). AMH is produced by granulosa cells of growing ovarian follicles and is commonly used to estimate ovarian reserve; lower concentrations generally indicate a smaller pool of recruitable follicles. However, the small observational study and the use of women with tubal-factor infertility rather than fertile women as controls preclude causal conclusions and limit generalisability to the wider population [97].

PVC has additionally been reported in renal and hepatobiliary specimens. Across kidney, adrenal gland, renal artery, renal vein and ureter samples from 28 kidney donors, PE and PVC were consistently the most abundant polymers by mass, although most associations with recipients’ early postoperative blood pressure were non-significant after correction for multiple testing [20]. MPs were also detected in all gallbladder and bile samples examined in a 23-sample surgical series, with PVC among the identified polymers but PE predominant [98].

Collectively, human studies document PVC in multiple biological specimens, as summarised in Table 1. Polymer identification, associations analysed separately for PVC, and associations involving aggregate MNP burden support different conclusions. Cardiovascular-event and vascular-severity associations based on MNP detection or total burden do not provide independent estimates of PVC effects. Separately analysed placental and reproductive associations remain subject to co-exposures, participant selection and observational design. Analytical interpretation also depends on contamination control, extraction recovery, polymer coverage and whether the endpoint represents particle abundance or polymer mass. These findings support occurrence in human specimens and hypothesis-generating associations; their evidential strength and inferential limits are considered in Section Evidence Strength and Inferential Limits.

### Evidence Strength and Inferential Limits

The strength of evidence depends on the conclusion being evaluated. The following framework distinguishes four categories of evidence and inference, while assessing analytical reliability and biological relevance within each category. These categories do not imply an automatic ranking of study designs: a controlled experiment may establish an effect in its model without establishing a corresponding human health risk. Confidence increases with adequate contamination controls and analytical validation, well-characterised test materials and doses, appropriate comparison groups, temporal ordering, dose-response information and consistency across independent studies [117,118].

Occurrence in biological specimens. Polymer identification or quantification supports the presence of PVC within the analysed specimen and analytical window. Confidence depends on procedural blanks, recovery, polymer specificity and size coverage. Detection alone does not establish the exposure source, route of entry, residence time, body-wide burden or a health effect.

Human observational associations. Analyses conducted separately for PVC can identify relationships with biomarkers or clinical endpoints. Their interpretation depends on participant selection, exposure measurement, control of confounding and temporal ordering. Cross-sectional associations remain vulnerable to reverse causation, and biomarkers are not interchangeable with incident disease. Prospective follow-up strengthens temporal interpretation, but an association involving aggregate MNP burden cannot be attributed independently to PVC.

Experimental effects and mechanisms. Controlled cellular and animal studies support effects of a characterised test material under defined exposure conditions. Mechanistic confidence increases when dose and time dependence are assessed and targeted interventions support the proposed pathway. Translation to humans additionally requires relevant exposure routes, delivered doses and endpoints. Without suitable particle and chemical controls, effects of PVC-containing material cannot be assigned solely to the polymer particle.

Human disease causation. This requires convergent evidence linking validated PVC exposure to subsequent clinically relevant outcomes, supported by compatible dose-response and mechanistic findings and adequate consideration of alternative explanations. No single detection result, biomarker association or animal experiment establishes this relationship.

Applied to the reviewed literature, evidence is most consistent for PVC occurrence in selected human specimens, although analytical heterogeneity prevents a harmonised estimate of internal burden. Evidence for PVC-related clinical associations remains limited and hypothesis-generating. Experimental studies provide model-dependent evidence of epithelial, oxidative and metabolic effects. Inflammatory findings are less consistent: cytokine responses in primary bronchial epithelial cells contrast with the absence of significant pro-inflammatory activation in macrophages exposed to fragmented PVC under the tested conditions [11,119]. Evidence remains insufficient to establish PVC-specific human disease causation or health-relevant internal-dose thresholds. Environmental studies inform material transformation and potential exposure pathways but do not directly establish human clinical effects.

## 6. Biological Effects of PVC-MNPs in Experimental Models

PVC-MNPs can exert biological effects beyond the disruption of anatomical barriers. At sites of first contact, local biological environments may modify particle properties before cellular interaction. In the gastrointestinal tract, these include digestive fluids, mucus, microbiota and surface-adsorbed biomolecules [10,120,121]. Cellular uptake, oxidative stress and metabolic alterations have also been reported in immune and stromal cell models [119,122,123]. Nominal culture concentrations and administered animal doses describe experimental inputs and should be distinguished from delivered and retained internal doses. Neither a “low” nor a “high” experimental dose establishes environmental or physiological relevance. Translation to human exposure requires comparable particle characteristics, exposure routes, dosimetry and duration. These experiments therefore inform hazard identification under the tested conditions, while human risk remains unresolved [117,118].

Studies using human intestinal cell models identify particle dimensions, inflammatory state and material conditioning as distinct considerations when comparing PVC-MNP responses. In Caco-2 cells, a human colorectal adenocarcinoma-derived intestinal epithelial model, PVC particles with a mean diameter of 136.5 µm were not internalised, whereas the smaller 1–4 µm PE fraction showed limited transepithelial transport. PVC reduced viability in Caco-2 cells, HepG2 hepatocellular carcinoma cells and differentiated HepaRG hepatocyte-like cells only at very high nominal concentrations, which the authors considered far above realistic dietary exposure [124]. The substantial size imbalance prevents attribution of the uptake differences to polymer identity, while these nominal culture concentrations cannot be directly equated with dietary intake.

Inflammatory state altered the response within an advanced intestinal model. In a triple culture combining Caco-2 cells, mucus-producing goblet-like HT29-MTX-E12 cells and macrophage-like cells derived from the human THP-1 monocytic line, PVC produced no measured acute effects under standard conditions but increased interleukin-1β (IL-1β) release and caused epithelial cell loss during experimentally induced inflammation [9]. This within-model contrast supports context-dependent susceptibility rather than a uniform intestinal response to PVC.

Material conditioning provides a further source of heterogeneity. In human induced pluripotent stem cell-derived intestinal epithelium, true-to-life PVC impaired barrier integrity and induced oxidative and cytokine responses. Similar categories of response occurred with PET containing titanium dioxide (TiO_2_), PP containing talc, and polyamide (PA), whereas PS did not produce the reported epithelial perturbations [10]. Responses correlated with protein corona composition, but this association does not establish the corona as the causal mediator. Together, these findings support biological activity of several formulated plastic materials while leaving the respective contributions of polymer chemistry, additives, particle properties and biological conditioning unresolved. A toxicity ranking based solely on polymer identity is therefore not supported.

Animal studies extend this evidence to the intestinal microenvironment, although their dosing regimens limit direct extrapolation to humans. In adult male mice, oral administration of 2 µm PVC particles at 100 mg per kg body weight (BW) per day for 60 days reduced mucus secretion, increased intestinal permeability and altered gut microbiota and faecal metabolomic profiles [125]. In female mice given aged or non-aged packaging-derived PVC particles by oral gavage at 0.02 mg/animal per dosing day, reported as 1 mg/kg BW, five days per week for 70 days, both particle types increased intestinal oxidative stress but produced different response profiles. Non-aged PVC induced a stronger pro-inflammatory response and greater reductions in short-chain fatty acids, whereas aged PVC caused more pronounced shortening of intestinal crypts, epithelial invaginations involved in tissue renewal, together with greater changes in mucus-secreting cells. Thus, ageing modified the pattern of intestinal toxicity rather than consistently increasing its severity [126]. Gastrointestinal transformation may also modify particle activity. Simulated digestion increased agglomeration, negative surface charge and protein corona formation on PVC, PET and PS NPs. Digested particles showed greater uptake and produced stronger epithelial disruption and inflammatory signalling in a co-culture of human Caco-2 cells and murine RAW264.7 macrophages than their non-digested counterparts [121]. This mixed-species model supports the importance of digestive conditioning but does not reproduce the complete human intestinal environment.

A complementary six-day intestinal tri-culture study compared aged and non-aged PVC MPs (50–100 µg/mL; median size 4–6 µm) with their bioaccessible fractions. Particle exposures increased paracellular permeability and induced oxidative and morphological changes, with several effects more pronounced for aged PVC. The bioaccessible fractions also produced toxicity, although generally to a lesser degree [127]. Considered alongside the endpoint-dependent ageing effects in the repeated-exposure mouse study, these findings support treating ageing as a change in particle properties and chemical exposure rather than a universal increase in toxicity. The activity of both preparations also leaves the relative contributions of particle-associated and released components unresolved.

Bronchial epithelial cultures represent the conducting airways, not the complete alveolar-capillary barrier, which also contains alveolar epithelial cells, basement membranes and capillary endothelial cells. Air–liquid interface (ALI) cultures are particularly relevant because particles are deposited onto the air-facing surface of the epithelium. In BEAS-2B cells, an immortalised human bronchial epithelial cell line, PVC NPs did not induce significant cell death or interleukin-8 (IL-8) release under ALI conditions [128]. A subsequent study using submerged BEAS-2B cultures likewise found no significant PVC-induced cytotoxicity or inflammatory response across the tested size fractions [129]. By contrast, in primary normal human bronchial epithelial cells exposed to nominal PVC doses of 0.003–0.100 µg/cm^2^, all three fractions (<1, 1–5 and 5–10 µm) produced modest increases in lactate dehydrogenase (LDH) release, indicating limited cell injury. Increased IL-8 and decreased monocyte chemoattractant protein-1 (MCP-1) secretion were observed, although statistical significance varied across doses and fractions [11]. In differentiated primary bronchial epithelial cultures obtained from three donors with chronic obstructive pulmonary disease (COPD) and three donors without COPD, PVC did not produce pronounced cytotoxicity or inflammatory responses at the group level. However, gene-expression responses involving antioxidant defence, apoptosis and autophagy varied between donors and clinical groups [130].

The divergent airway findings require consideration of epithelial origin and differentiation, donor background, particle conditioning and exposure method. Because these factors vary between studies, the available comparisons do not isolate their respective contributions to the differences in response [11,129,130]. An informative component comparison was provided by Staal et al.: PVC leachate did not reproduce the overall pattern of cell injury and cytokine changes observed with the particle-containing exposures [11]. However, the leachate was collected during particle production in 1-propanol and therefore does not represent chemical release into physiological airway fluids. This comparison supports a contribution associated with the particle-containing exposure under the tested conditions, while leaving the active component unresolved. It cannot by itself distinguish effects of the PVC polymer from those of particle-bound constituents or chemicals released subsequently during biological contact.

Additional pulmonary models identify oxidative and metabolic changes that are not equivalent to disruption of an epithelial barrier. PVC induced senescence-associated changes in A549 human lung adenocarcinoma-derived epithelial cells and BEAS-2B bronchial epithelial cells. In A549 cells, PVC increased ROS production, and antioxidant treatment reduced the senescent phenotype, supporting the involvement of oxidative stress in this model [14]. Model PVC NPs measuring approximately 120–140 nm were also internalised by IMR-90 cells, a normal human foetal lung fibroblast line. Exposure reduced cell viability and ATP production, increased ROS formation and LDH leakage, and induced cell-cycle arrest and apoptosis [122]. These findings concern epithelial and stromal cell responses and do not, by themselves, demonstrate injury to the complete alveolar-capillary barrier.

The available human macrophage experiments used macrophage-like cells differentiated from the human THP-1 monocytic cell line rather than primary macrophages isolated from donors. Mechanically fragmented PVC reduced mitochondrial activity and increased LDH leakage at higher experimental concentrations but did not significantly activate nuclear factor kappa B (NF-κB) or increase the secretion of IL-6, IL-1β or tumour necrosis factor-α [119]. In a separate study, THP-1 cells were differentiated into macrophage-like cells and experimentally polarised towards a pro-inflammatory phenotype resembling that of classically activated macrophages (M1-like). Labelled model PVC NPs altered cell morphology and lipid-droplet accumulation without significantly reducing cell viability under the tested conditions [123]. Thus, intracellular accumulation and metabolic disturbance may occur without a measurable pro-inflammatory cytokine response.

Evidence from ocular models remains limited. In a transformed human corneal epithelial cell line, both PS and PVC particles were internalised and induced cytotoxic and inflammatory responses associated with mitochondrial damage and disrupted lipid metabolism. Topical administration also caused dry eye-like changes in mice, although this does not establish comparable effects under environmentally relevant human ocular exposure [131].

Evidence from ovarian cell models is even more limited. Labelled model PVC NPs were detected within SK-OV-3 human ovarian cancer cells [132]. This finding confirms particle internalisation by a malignant ovarian cell line but does not demonstrate effects on non-malignant ovarian cells, ovarian function or fertility.

Direct evidence of interactions between PVC-MNPs and human skin remains scarce, and current interpretation relies mainly on studies of other polymers. Across dermal uptake studies, particles were usually retained within the stratum corneum or hair follicles, whereas penetration into viable epidermal and dermal tissue was limited and strongly dependent on skin condition and experimental method. Most of this evidence concerned PS rather than PVC [72]. In ex vivo human skin, an intact stratum corneum largely retained 100 nm PS particles at the skin surface, while its removal markedly increased particle penetration throughout the epidermis, with only limited entry into the dermis. In complementary models lacking a normal stratum corneum, the particles were internalised by keratinocytes and reached dermal fibroblasts, where they induced inflammatory and tissue-remodelling responses. These findings identify skin integrity as an important determinant of nanoplastic penetration but cannot be extrapolated to PVC-MNPs or used as evidence of their penetration through intact human skin [73].

Exposure dose and duration should be considered separately when interpreting pathological mechanisms. Acute exposure at high experimental PVC concentrations can reveal oxidative stress, impaired ATP production, membrane damage and apoptotic cell death [119,122]. These findings characterise cellular injury under the tested conditions but do not establish the mechanisms operating during chronic low-dose exposure. Inflammatory activation is also not inevitable: fragmented PVC reduced metabolic activity and increased LDH leakage in THP-1-derived macrophages without significant NF-κB activation or pro-inflammatory cytokine induction [119]. Conversely, acute changes in LDH release and cytokine secretion have been reported at nominal surface doses of 0.003–0.100 µg/cm^2^ in primary bronchial epithelial cultures [11]. Acute effects therefore cannot be attributed solely to high dose, and their interpretation also depends on the model, particle properties and delivery method.

For repeated exposure at lower administered doses, a central mechanistic question is whether sublethal perturbations produce sustained changes in redox balance, epithelial maintenance, microbiota metabolism and immune regulation without immediate widespread cell death. Repeated-exposure PVC studies support the investigation of these processes. The 70-day mouse study using 0.02 mg/animal on dosing days, five days per week, reported intestinal oxidative stress, inflammatory changes, altered crypt morphology and reduced short-chain fatty acids [126]. In a separate 26-day dietary study, environmentally sourced PVC increased colonic inflammatory and oxidative-stress markers without overt histomorphological changes under steady-state conditions. In mice with experimentally induced chronic colitis, however, PVC exacerbated inflammation, tissue damage and fibrosis-related responses [28]. These findings indicate that repeated exposure can alter tissue homeostasis and interact with pre-existing inflammation, although they do not establish equivalent effects at human environmental doses.

Oxidative and inflammatory processes may overlap across exposure regimens; potential differences concern their time course, persistence and functional consequences. The repeated-exposure studies discussed here do not establish mechanisms of chronic low-dose toxicity in humans or define a PVC-specific environmentally relevant internal dose. Resolving this gap requires prolonged low-dose experiments with measurements of delivered or retained dose, serial assessment of relevant functional endpoints, and recovery periods after exposure cessation. Such designs would help distinguish transient adaptive responses from persistent dysfunction and progressive tissue injury.

Table 2 compares selected experimental PVC exposure conditions with published, source-specific estimates of external human exposure through drinking water, indoor dust and indoor air [59,62,63]. The comparison uses consistent dimensions: exposure route or model, particle characteristics, dose metric, exposure duration and the principal limitation of extrapolation to humans. Administered animal doses and nominal culture concentrations describe experimental inputs. Delivered dose is the amount reaching a specified biological target, whereas measured internal burden reflects material present in a sampled compartment and is influenced by uptake, distribution and clearance. The human estimates concern mixed-polymer MPs rather than PVC-specific absorbed or retained dose.

The comparison identifies distinct limitations across exposure models. The oral mouse studies report body-weight-normalised doses of 100 and 1 mg/kg BW on dosing days, respectively [125,126], whereas the selected human exposure estimates are expressed as numbers of mixed-polymer particles [59,62,63]. Differences in polymer composition, particle-size coverage and exposure schedules prevent direct quantitative alignment. In submerged macrophages, the modelled deposition of approximately 95% of administered PVC mass describes delivery within the culture system, not absorption following human ingestion [119]. The ALI study reports nominal surface doses of 0.003–0.100 µg/cm^2^, but actual deposition was not quantified and the 24-h endpoint does not represent chronic retention [11]. Quantitative extrapolation requires alignment of particle characteristics and exposure scenarios, together with information on deposition, absorption, distribution and clearance [67]. Within the framework in Section Evidence Strength and Inferential Limits, these studies support experimental hazard and biological plausibility, while the magnitude of risk at human exposure levels remains unresolved.

## 7. Environmental Fate and Biological Effects of PVC-MNPs in a One Health Context

A One Health interpretation of PVC-MNPs should connect shared exposure sources and transformations with environmental, animal and human outcomes. We distinguish three overlapping pathways: movement of PVC-containing particles through environmental compartments and food systems; release and redistribution of associated chemicals; and microbial colonisation or chemical-driven changes in microbial communities. PVC detection in edible seafood, ageing-dependent chemical release, microbial responses to PVC leachate and experimental pathogen transmission to shrimp provide evidence for selected links within these pathways [25,60,133,134]. Animal health and ecological functions are relevant endpoints in their own right. Assessing their implications for human health also requires evidence of transfer, human exposure and biological effects. Existing studies do not establish a complete pathway from the exposure source to human disease.

Aquatic food systems provide the most direct connection with dietary exposure. A systematic review documented MPs in fish, crustaceans and molluscs, but high heterogeneity in fish and shellfish datasets limited quantitative interpretation [135]. In a recirculating aquaculture system, particles were recovered from water, feed and tissues of all 55 European seabass, with higher concentrations in the gastrointestinal tract and gills than in muscle. Man-made cellulose/rayon and PET fibres predominated in water and fish, while PE, PP and PVC associated with system components contributed little to the overall profile. Water and feed were therefore identified as the principal particle sources, but the low contribution of PVC provides only limited evidence for PVC-specific transfer through farmed fish [136].

Environmental contamination may therefore reach humans through uptake by aquatic organisms, possible transfer from prey to predators, and consumption of contaminated edible portions. PVC polymer mass has been quantified in edible seafood, supporting a potential oral exposure pathway, although contributions from handling and packaging prevent attribution solely to environmental uptake [60]. Following ingestion, PVC-containing material may interact with intestinal mucus and epithelium, providing a plausible connection with the barrier and oxidative responses discussed in Section 6. Trophic transfer should nevertheless be distinguished from bioaccumulation, which involves net retention within an organism, and biomagnification, which involves increasing concentrations across trophic levels. The occurrence data discussed here do not establish PVC-specific bioaccumulation or biomagnification along the food chain [60,136]. The chemical vector role of PVC also requires comparison with the surrounding exposure matrix. In equilibrium experiments using diet and faecal samples from a captive polar bear, added PVC MPs slightly reduced the fugacities of spiked polychlorinated biphenyls but substantially increased those of the polymer additives UV-328 and octabenzone [137]. These equilibrium measurements concern the potential for chemical uptake rather than measured absorption in a living animal, and the PVC concentrations exceeded current observations. The findings nevertheless show why particle presence alone cannot establish enhanced chemical exposure: the direction and magnitude of the effect depend on the compound, particle contamination and receiving matrix.

Mechanically fragmented PVC MPs produced from plumbing pipe were tested in two animal models: the brittle star *Ophiactis virens* during arm regeneration and larvae of the amphibian *Xenopus laevis*. No adverse effects were detected in either model at 0.1 µg/mL. At 1 µg/mL, PVC reduced tissue differentiation during arm regeneration in *O. virens*, whereas intestinal degeneration and altered swimming behaviour in *X. laevis* larvae occurred at 10 µg/mL [138]. These organism- and concentration-specific responses cannot be translated directly into human-health effects.

Microbial effects extend this context beyond direct particle toxicity. In carp exposed for eight weeks, PVC-MPs, alone or combined with oxytetracycline or sulfamethazine, enriched potential pathogens, including *Enterobacter* and *Acinetobacter*, and altered intestinal antibiotic resistance gene (ARG) profiles. Co-exposure to PVC and oxytetracycline increased tetracycline-resistance genes 1.40-fold relative to oxytetracycline alone, although total ARG and mobile genetic element abundance remained below control levels [139]. PVC therefore changed the distribution of intestinal resistance markers rather than uniformly increasing them. In a seawater microcosm, particle-free PVC leachate enriched genes associated with multidrug, aminoglycoside and peptide antibiotic resistance, together with genes involved in virulence-protein secretion [133].

Environmental microbial conditioning is not limited to aquatic animals. In wheat rhizosphere soil, 200 µm PE, PVC and PS microplastics added at 2% by weight produced polymer-dependent changes in bacterial diversity, community composition and predicted functional profiles. PE and PVC increased predicted pathways related to metabolism and xenobiotic biodegradation [140]. Functional measurements provide a complementary perspective. In a 60-day soil experiment, plasticiser-containing PVC altered nitrogen pools, microbial communities and nitrogen-cycling processes. Exposure to the phthalate plasticiser alone produced similar changes in soil nitrogen parameters, whereas pure PVC polymer preparations had little effect [141]. These comparisons implicate released additives as an important driver in the tested system and connect the particle-chemical framework with ecosystem functions relevant to soil and plant health. Plastic particles may also be redistributed between ecosystems by animals. A study of five seabird species in northern Chile examined their role as biovectors, meaning organisms that transport material across ecosystem boundaries. Plastics were detected at nesting colonies and in the stomach contents of selected species, but their overall transfer from sea to land was limited; the clearest evidence concerned macroplastics transported to kelp-gull colonies through regurgitation rather than PVC-specific microplastic transport [142]. In coral areas, biofilm communities formed on nine types of microplastics differed from those in the surrounding seawater, and their composition depended on both polymer type and deployment site [143]. These findings show that environmental context can influence the microbial associations and spatial redistribution of plastic particles, but do not establish subsequent human exposure or health effects.

Microbial colonisation provides a further potential link to human exposure. In an experimental aquaculture study, PVC MPs colonised by *Vibrio parahaemolyticus* facilitated bacterial transmission from surrounding water to the shrimp *Litopenaeus vannamei*, with accompanying hepatopancreatic damage [134]. This provides a PVC-specific example of a microbial carrier pathway in an aquaculture species; transmission to human consumers was not examined.

If viable human pathogens or resistant bacteria associated with plastic biofilms enter food or water consumed by humans, they could provide an additional microbial exposure route. However, microbial community changes and resistance-gene findings in fish guts, PVC-leachate-exposed communities and environmental biofilms do not establish transmission to consumers [133,139,143]. Establishing a human-health consequence would require characterisation of microbial viability and human-relevant pathogenic or resistance traits. It would also require evidence of survival through relevant food processing or water treatment and gastrointestinal passage, and exposure sufficient to cause colonisation, infection or resistance-gene transfer. ARG detection or enrichment alone does not demonstrate phenotypic antibiotic resistance or transfer to the human gut microbiota.

Biological processing does not imply complete removal. In *Tenebrio molitor* larvae fed rigid PVC MPs, the weight-, number- and size-average molecular weights of the residual polymer decreased by 33.4%, 32.8% and 36.4%, respectively, while chloride release represented approximately 2.9% of ingested PVC; suppression of the gut microbiota with gentamicin markedly inhibited depolymerisation [54]. *Zophobas atratus* larvae achieved a reported PVC removal efficiency of 40.5%, accompanied by partial depolymerisation, chloride release and the formation of chlorinated organic intermediates; PVC feeding also increased gut oxidative stress [144]. Environmental organisms can therefore modify the dimensions, chemistry and biological identity of PVC-MNPs without completely mineralising the polymer. From a One Health perspective, these processes may alter the form in which PVC-MNPs circulate between ecosystems and potentially reach food systems or human biological interfaces, rather than eliminating their biological relevance.

An operational One Health research agenda should examine these connections within the same exposure system. For an aquatic food pathway, this could pair measurements in source water and feed with analyses of animal tissues and edible portions before and after handling or processing, helping distinguish environmental uptake from subsequent contamination [60]. Measurements should cover particle identity and size distribution, associated chemicals and their bioaccessibility, and microbial viability or function where relevant to the pathway [133,134]. Linking these observations to characterised human intake would provide a stronger basis for exposure assessment than extrapolating directly from ecotoxicological responses. Potential interventions should likewise be evaluated across the relevant exposure components: reduced particle abundance alone would not demonstrate reduced chemical exposure or microbial hazard. Similarly, fragmentation or partial depolymerisation should not be treated as detoxification without assessing residual particles and transformation products.

## 8. Future Directions and Conclusions

Current evidence identifies three principal research gaps. First, source-resolved exposure assessment remains limited. Polymer identity alone does not capture product origin, formulation, ageing state or the mobile chemical fraction associated with the particles. Substantial uncertainty also persists regarding exposure dose, biological persistence and target-tissue burden [118,145].

Second, mechanistic attribution remains limited when experiments do not separate the particulate material from its released chemical fraction. The component comparisons and material-dependent responses discussed in Section 6 demonstrate why this distinction matters [10,11,129]. We propose a comparative design using material from the same parent batch: the original particle-containing preparation, a particle-free leachate, washed particles, and a reconstituted particle-leachate preparation, together with matched medium, solvent and procedural controls. Particle removal from leachates should be verified within stated analytical detection limits, and chemical release should be characterised in media and over durations relevant to the intended exposure. Washed particles require renewed physicochemical characterisation because washing may alter both chemical content and surface properties. These preparations should therefore be treated as operational experimental contrasts, with particle and chemical doses reported separately.

The purpose of this design is to determine which exposure component contributes to a response and whether combining particles with leachate changes that response. A greater response to the combined preparation than to particles alone is insufficient to establish synergy; this requires comparison with a specified expectation for combined effects. Cross-polymer experiments should also control particle dimensions, morphology and delivered dose as far as feasible, while reporting remaining differences in formulation and biological conditioning. Route-relevant models, repeated exposure and recovery measurements would then help determine whether the identified responses are transient, persistent or dependent on pre-existing inflammation. These priorities follow from our synthesis of the evidence rather than an assumption that all PVC materials share a single toxicity mechanism.

Third, methodological comparability remains constrained by incomplete reporting of QA/QC, particle-size detection limits, polymer-identification procedures, sample preparation, contamination controls, analytical recovery and the reporting of particle counts, morphology and polymer mass [83,117]. This heterogeneity limits cross-study comparison and prevents reliable quantitative assessment of human-health risk.

Overall, PVC-MNPs represent heterogeneous particle-chemical exposures. The qualitative appraisal in Section Evidence Strength and Inferential Limits identifies more consistent support for occurrence in human specimens than for PVC-related clinical effects. Human associations remain observational, whereas experiments support hazard identification and biological plausibility under defined conditions. Current evidence does not establish PVC-specific human disease causation or health-relevant exposure thresholds. The main challenge is to link characterised external exposure, delivered and retained internal doses, and clinically relevant outcomes [67,118].

Priority should therefore be given to large-scale, prospective epidemiological cohort studies that link validated measurements of internal PVC exposure with incident clinical disease. These studies should include diverse populations and repeated PVC-specific measurements in accessible biological samples collected before the outcomes of interest occur. Analytical and QA/QC procedures should be harmonised in line with the reporting framework proposed in Section 5. Follow-up should be sufficiently long to capture clinically verified outcomes, with careful assessment of baseline health, established disease risk factors and relevant co-exposures. Such designs would help assess temporal ordering and exposure-response relationships and reduce, although not eliminate, concerns about reverse causation. Interpreted alongside mechanistic evidence, their findings could strengthen causal inference and help bridge the gap between internal exposure and clinical disease. Residual confounding and exposure-measurement uncertainty should nevertheless remain explicit considerations.

## Figures and Tables

**Table 1:** Human biological specimens and clinical observations relevant to PVC biomonitoring. PVC evidence includes polymer detection and quantification, and associations analysed separately for PVC. Broader context covers findings involving other polymers, total MNP burden, PVC-associated additives and animal studies. Detection does not establish causality, and methodological differences limit comparisons across studies. Abbreviations: BALF, bronchoalveolar lavage fluid; CD11b, cluster of differentiation 11b; CNS, central nervous system; CSF, cerebrospinal fluid; MNPs, micro- and nanoplastics; MPs, microplastics; PA66, polyamide 66; PE, polyethylene; PP, polypropylene; PVC, polyvinyl chloride.

Biological Sample or Specimen	Anatomical or Clinical Relevance	PVC Evidence and Broader Context	Principal Limitation	Citations
**Blood**	Circulating plastic burden and possible direct input from medical procedures	PVC has been identified or quantified in blood. Higher PVC concentrations have been associated with higher eosinophil CD11b expression. Associations involving total MNP burden provide broader vascular and immune context	Does not identify the exposure source or tissue distribution; results are highly dependent on collection, contamination control, recovery and analytical method	PVC evidence: [84,99]; Broader context: [84,99]
**Faeces**	Material passing through the gastrointestinal tract, including ingested particles that were not absorbed	PVC has been identified or quantified in faeces, consistent with gastrointestinal exposure and passage. Stool measurements do not establish intestinal absorption or systemic transfer	Strongly affected by diet, gastrointestinal transit, sample heterogeneity, collection procedure and external contamination	PVC evidence: [86,100,101]
**Urine**	Possible urinary elimination of sufficiently small particles or polymer-associated material	PVC has been identified in urine. An exploratory analysis suggested a positive association between PVC and glycaemic markers at higher urinary concentrations. The main adjusted associations concerned polyamide, polypropylene and the combined MP mixture. The pathway and kinetics of urinary PVC elimination remain uncertain	Entry of particles into urine remains poorly understood, and the available evidence is limited	PVC evidence: [102,103]; Broader context: [103]
**Nasal lavage, sputum**	Material deposited in or cleared from the respiratory tract	PVC has been identified in nasal lavage and sputum. Detection supports respiratory exposure but does not quantify mucociliary clearance or establish a site of tissue retention	Contains material from different airway regions and does not establish retention within lung tissue	PVC evidence: [17,104]
**BALF, lung tissue**	Material present in bronchiolar and alveolar compartments or retained within pulmonary tissue	PVC has been identified in BALF and lung tissue. Reported inflammatory, imaging and blood-test associations concern overall particle measures rather than PVC-specific burden	BALF requires bronchoscopy and samples airway-lining material rather than tissue; lung tissue is usually obtained from clinically selected surgical or post-mortem cases	PVC evidence in BALF: [90]; PVC evidence in lung tissue: [105,106]; Broader context: [90,106]
**Vascular plaques, arterial tissues, thrombi**	Local particle burden in diseased vascular lesions and thrombotic material	PVC has been identified or quantified in vascular specimens. Reported cardiovascular-event and thrombus-severity associations concern MNP detection or total MP burden rather than independently estimated PVC effects	Clinically selected observational specimens cannot distinguish whether particle accumulation is a cause, consequence or accompanying feature of vascular disease	PVC evidence: [21,81,85,107]; Broader context: [21,81]
**Placenta, amniotic fluid**	Exposure-related material at the maternal-foetal interface and within the intra-amniotic compartment	PVC has been identified or quantified in placental tissue. Associations analysed separately for placental PVC include telomere length in placental tissue and cord blood, cord-blood steroid hormone concentrations and cord-blood alkaline phosphatase activity. Amniotic-fluid findings provide broader context involving other polymers and a PVC-associated stabiliser; stabiliser detection does not establish PVC particle identity	Susceptible to contamination and tissue heterogeneity; detection does not demonstrate foetal transfer, developmental toxicity or involvement in pregnancy outcomes	PVC evidence: [91,92,93,94]; Broader context: [108]
**Umbilical cord blood**	Foetal-side circulation at delivery	PVC has been identified in umbilical cord blood, documenting occurrence in the foetal-side circulation at delivery. Detection does not establish the exposure source, timing or route of entry	Highly sensitive to contamination; the source, timing and route of particle entry remain uncertain	PVC evidence: [109]
**Breast milk**	Potential route of postnatal oral exposure during lactation	PVC has been identified in breast milk, indicating a potential route of infant oral exposure. Reported microbiota associations concern samples with and without MPs rather than PVC separately	The lipid-rich composition complicates analysis, while collection, storage and container contamination can influence results	PVC evidence: [110,111]; Broader context: [111]
**Follicular fluid, seminal plasma, semen, testis**	Fluid and tissue compartments directly associated with gametes and reproductive function	PVC has been identified or quantified in human reproductive fluids and testes. Selected human studies report associations analysed separately for PVC with fertilisation, sperm motility or ovarian reserve. The larger follicular-fluid study also reported higher PVC concentrations in the diminished-ovarian-reserve group, although its principal focus was PA66. Testis-weight associations in the human/canine study were evaluated in dogs, and mechanistic findings from studies of other polymers or MNP mixtures should not be attributed to PVC	Participant selection, comparison groups and reproductive endpoints vary across studies. Observational associations do not establish reproductive causality, and canine findings require separate interpretation from human observations.	PVC evidence: [19,95,96,97,112,113]; Broader context: [19,113]
**CSF, CNS tissue**	Fluid surrounding the brain and spinal cord or tissue from the central nervous system	PVC has been identified or quantified in CSF and brain tissue. In one observational study, CSF PVC concentrations were higher in amyloid-positive than in amyloid-negative participants. Reported cognitive associations concerned PE, metabolic findings concerned total MNP burden, and albumin-index associations concerned PE and PP. These findings do not establish a PVC-specific causal effect on neurological disease	CSF does not directly demonstrate deposition in brain tissue; samples are invasive or post-mortem and may be affected by neurological disease or barrier disruption	PVC evidence: [88,89,114,115]; Broader context: [88,89,114]
**Bone marrow**	Possible internal particle burden in haematopoietic tissue	PVC has been quantified in bone marrow, extending evidence of internal occurrence. The available study does not establish a PVC-specific haematological effect or a health-relevant exposure threshold	Evidence remains scarce; sampling is invasive and matrix-specific analytical recovery is uncertain	PVC evidence: [87]
**Kidney, gallbladder, bile and post-mortem organs**	Renal, hepatobiliary and other internal tissue compartments	PVC has been identified or quantified in renal and hepatobiliary specimens and in post-mortem organ specimens. These findings do not establish organ-specific injury or PVC-specific elimination pathways	Surgical, donor and post-mortem specimens represent selected populations. The post-mortem organ study involved a single donor; polymer identity and size-fraction findings must be interpreted for each specimen separately	PVC evidence: [20,98,116]

**Table 2:** Selected experimental PVC exposure conditions and estimates of external human microplastic exposure: dose metrics and limits of comparability. Experimental doses and human exposure estimates are presented using the metrics reported in the original studies. Human estimates concern mixed-polymer MPs rather than PVC-specific absorbed doses. Differences in dose metrics, particle characteristics and exposure routes prevent direct comparisons or determination of human-equivalent doses and health thresholds. Abbreviations: ALI, air–liquid interface; BW, body weight; MNPs, micro- and nanoplastics; MPs, microplastics; PET, polyethylene terephthalate; PVC, polyvinyl chloride.

Model or Exposure Scenario	Material and Particle Characteristics	Reported Dose or Exposure Estimate	Main Limitation for Comparison with Human Exposure	Ref.
**A. Experimental PVC exposure conditions**				
**Adult male mice; oral gavage**	PVC MPs; 2 µm	100 mg/kg BW/day; once daily for 60 days	Repeated bolus administration. Translation to human exposure requires information on absorption, retention and differences in exposure pattern	[125]
**Female BALB/c mice; oral gavage**	Packaging-derived PVC MPs; measured size ranges: non-aged, 1.3–31.4 µm; aged, 1.2–26.4 µm	0.02 mg/animal per dosing day; five days per week for 70 days	Repeated bolus gavage. Packaging origin and the lower administered dose do not establish equivalence to human PVC intake or target-tissue exposure	[126]
**THP-1-derived macrophages; submerged culture**	Mechanically fragmented PVC; <1 µm and 1–5 µm fractions	Nominal concentrations of 1, 10 and 100 µg/mL; 24 h	Dosimetry modelling predicted deposition of approximately 95% of administered PVC mass within 24 h. Direct macrophage exposure bypasses epithelial entry barriers	[119]
**Primary human bronchial epithelial cells; ALI nebulisation**	PVC; <1 µm, 1–5 µm and 5–10 µm fractions	Nominal surface doses of 0.003, 0.017 and 0.100 µg/cm^2^; endpoints assessed 24 h after exposure	Actual deposited dose could not be quantified. A single exposure with an acute endpoint does not reproduce long-term airway deposition and clearance	[11]
**B. Estimated external human exposure to mixed-polymer MPs**				
**Drinking-water ingestion; estimates for the United Kingdom**	Mixed-polymer MPs; PVC among the common polymers detected in tap water	Adults: 1 MP/kg BW/day; infants/toddlers: 4 MPs/kg BW/day	Estimates based on measured water concentrations and assumed consumption. They do not quantify PVC-specific intake or absorbed dose	[59]
**Inadvertent ingestion of indoor dust; Birmingham, United Kingdom**	Mixed-polymer MPs, including PVC; analysed size range: 10–150 µm	Adults: 0.8 MPs/kg BW/day; toddlers: 9.7 MPs/kg BW/day	Source-specific ingestion estimates for the analysed size fraction. They do not establish exposure to the smaller fractions used in some experiments	[62]
**I** **ndoor-air inhalation; Birmingham, United Kingdom**	Mixed-polymer MPs; PET and PVC prominent; airborne particles predominantly in the 10–25 µm size class	Adults: 3.0 MPs/kg BW/day; toddlers: 6.3 MPs/kg BW/day	Estimated inhaled particle numbers. Size-dependent deposition and clearance determine the fraction reaching and remaining at biological targets	[63]

## Data Availability

No new data were created or analysed in this study. Data sharing is not applicable to this article.

## Abbreviations

The following abbreviations are used in this manuscript:

ALI	air–liquid interface
ALP	alkaline phosphatase
ALT	alanine aminotransferase
AMH	anti-Müllerian hormone
ARG	antibiotic resistance gene
AST	aspartate aminotransferase
ATP	adenosine triphosphate
BALF	bronchoalveolar lavage fluid
BW	body weight
CD11b	cluster of differentiation 11b
CNS	central nervous system
COPD	chronic obstructive pulmonary disease
CSF	cerebrospinal fluid
DBP	dibutyl phthalate
DEHP	di(2-ethylhexyl) phthalate
DOR	diminished ovarian reserve
GGT	gamma-glutamyl transferase
IL-1β	interleukin-1β
IL-6	interleukin-6
IL-8	interleukin-8
IL-12p70	interleukin-12p70
LDH	lactate dehydrogenase
LDIR	laser direct infrared spectroscopy
M1	classically activated macrophage phenotype
MCP-1	monocyte chemoattractant protein-1
MEHP	mono(2-ethylhexyl) phthalate
µFTIR	micro-Fourier transform infrared spectroscopy
MMSE	Mini-Mental State Examination
MNPs	micro- and nanoplastics
MPs	microplastics
NF-κB	nuclear factor kappa B
NK	natural killer
NPs	nanoplastics
PA	polyamide
PA66	polyamide 66
PE	polyethylene
PET	polyethylene terephthalate
PP	polypropylene
PS	polystyrene
PVC	polyvinyl chloride
PVC-MNPs	polyvinyl chloride micro- and nanoplastics
PVC-MPs	polyvinyl chloride microplastics
PVC-NPs	polyvinyl chloride nanoplastics
Py-GC/MS	pyrolysis–gas chromatography–mass spectrometry
QA/QC	quality assurance and quality control
ROS	reactive oxygen species
UV	ultraviolet
WoS	Web of Science
